# Impact of Different Cell Types on the Osteogenic Differentiation Process of Mesenchymal Stem Cells

**DOI:** 10.1155/sci/5551222

**Published:** 2025-02-13

**Authors:** Zixin Wang, Lina Ren, Zhengtao Li, Qingyuan Qiu, Haonan Wang, Xin Huang, Dongyang Ma

**Affiliations:** ^1^School of Stomatology, Lanzhou University, Lanzhou, China; ^2^Department of Oral and Maxillofacial Surgery, The 940th Hospital of Joint Logistics Support Force of PLA, Lanzhou, China

**Keywords:** bone healing, mesenchymal stem cell, osteogenic differentiation, tissue engineering

## Abstract

The skeleton is an important organ in the human body. Bone defects caused by trauma, inflammation, tumors, and other reasons can impact the quality of life of patients. Although the skeleton has a certain ability to repair itself, the current most effective method is still autologous bone transplantation due to factors such as blood supply and defect size. Modern medicine is attempting to overcome these limitations through cell therapy, with mesenchymal stem cells (MSCs) playing a crucial role. MSCs can be extracted from different tissues, and their differentiation potential varies depending on the source. Various cells and cell secretions can influence this process. This article, based on previous research, reviews the effects of macrophages, endothelial cells (ECs), nerve cells, periodontal cells, and even some bacteria on MSC osteogenic differentiation, aiming to provide a reference for multicell coculture strategies related to osteogenesis.

## 1. Introduction

Bone constitutes a crucial component of the human body, endowed with a certain capacity for repair and regeneration. However, substantial challenges arise in achieving optimal bone repair, particularly in cases of extensive bone defects or severe fracture dislocations encountered in clinical practice. In such instances, effective medical intervention becomes imperative. The process of bone repair is intricate, involving a myriad of cells. Natural bone comprises a diverse array of cell types, encompassing mesenchymal stem cells (MSCs), fibroblasts, endothelial cells (ECs), nerve cells, osteoblasts, and osteoclasts (OCs), among others. The original bone serves as both the anchor point for calcium salt deposition and the scaffold for cellular mass, constituting a pivotal element in the reparative process. Various bone repair methods, including autologous bone grafting [1], distraction osteogenesis [2], osteoinductive membrane technology [3], cell sheet technology [4], and artificial biomaterial implantation, continue to face limitations such as suboptimal reconstruction effects, restricted donor bone sources, and a lack of biological functionality. Consequently, leveraging bone tissue engineering technology as a functional repair approach emerges as an ideal strategy for addressing bone defects. Among the avenues of investigation, cell therapy stands out as a prominent focus. MSCs, distinguished by their ethical feasibility, robust regenerative properties, versatile differentiation capabilities, and wide availability, have gained significant traction in both scientific and clinical research. As integral contributors to bone tissue engineering, MSCs offer a promising avenue for effective bone defect repair.

MSCs are a type of unspecialized adult stem cells (ASCs) known for their remarkable capacity for multidirectional differentiation [5, 6]. The Mesenchymal and Tissue Stem Cell Committee of the International Society for Cell Therapy has outlined the minimum criteria that MSCs must meet, including the following:

1. Plastic adherence: MSCs must exhibit plastic adherence under standard culture conditions.
2. Surface marker expression: The expression rates of positive markers CD105, CD73, and CD90 on most MSCs should be ≥95%, while the expression rates of negative markers CD45, CD34, CD14 or CD11b, CD79a or CD19, and HLA-DR should be ≤2%.
3. In vitro differentiation: MSCs must demonstrate the ability to differentiate into osteoblasts, adipocytes, and chondroblasts in vitro [7].

MSCs are derived from a diverse array of sources, reflecting the richness of available options. Commencing with the earliest isolation from bone marrow (BM-MSCs), scholars have progressively extracted MSCs from various tissues, including adipose tissue (AT-MSCs), teeth, amniotic fluid, umbilical cord (UC-MSCs), UC Wharton's Jelly (WJ-MSCs), placenta (PL-MSCs), liver, tendon, urine, peripheral blood, dermis, gingiva, and heart [8]. Dental- and periodontal-derived MSCs encompass periodontal ligament stem cells (PDLSCs), gingival stem cells (GMSCs), dental follicle stem cells (DFSCs), dental pulp stem cells (DPSCs), human deciduous pulp stem cells (SHEDs), and apical stem cells (SCAPs). To date, BM-MSCs and AT-MSCs have been the most extensively studied among these diverse sources.

From the perspective of bone development, both intramembranous and endochondral ossifications are influenced by various types of cells throughout the process. Firstly, the stem cells involved in intramembranous ossification primarily originate from the neuroectoderm, while those in endochondral ossifications predominantly derive from the paraxial mesoderm and lateral plate mesoderm. This difference in stem cell origin leads to variations in their osteogenic potential. Additionally, the development of the vascular, neural, and immune systems during the ossification process plays a crucial role in the migration, proliferation, and differentiation of stem cells [9]. From the perspective of bone repair following injury, when bone damage occurs, local blood vessels experience rupture, leading to congestion. Inflammatory cells, notably macrophages, swiftly arrive at the injury site to activate inflammation, releasing inflammatory factors and attracting ECs and MSCs [10, 11]. Simultaneously, blood vessels and nerves infiltrate the injured area, providing essential nutrients and guidance, thereby facilitating the homing, proliferation, and differentiation of stem cells. MSCs, sourced from the periosteum or adjacent regions, respond to the local microenvironment by initiating proliferation and differentiation [12]. In the wake of bone injury, osteoblasts and OCs within the primary bone tissue undergo natural apoptosis or perish, contributing cellular contents or apoptotic bodies (ABs) that play a role in the inflammatory process and influence the differentiation of MSCs. The impact of bacteria primarily manifests in the early stages of inflammation repair, exacerbating inflammation and stimulating the aggregation of inflammatory cells, thereby promoting the proliferation of EC and MSCs. As the macrophages transition from the M1 to the M2 phenotype, local inflammation gradually subsides, and the behavior of stem cells shifts from proliferation to differentiation. MSCs progressively differentiate from preosteoblasts into osteoblasts, leading to the gradual accumulation of secreted calcium salts and eventual completion of ossification. The equilibrium between osteogenesis and OC activity is re-established, facilitating the integration and repair of the new bone with the original bone over subsequent periods. In summary, various cells play a crucial role in the proliferation and differentiation of MSCs during the natural processes of bone formation and repair. This review aims to delineate the effects of various cells and/or cell products on the osteogenic differentiation of MSCs, elucidating the pathways involved. Such insights contribute to a deeper understanding of MSC osteogenesis and offer valuable reference points for the development of strategies involving multicell coculture to promote osteogenesis.

## 2. Osteogenic Differentiation of MSCs

### 2.1. The Process of Osteogenic Differentiation

**Figure 1:**
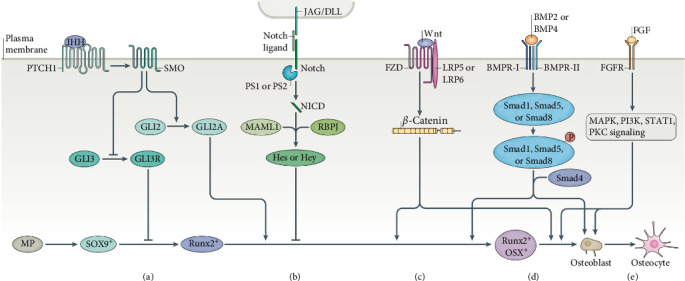
Developmental signaling pathways regulating osteoblast differentiation. Various signaling pathways function in a coordinated manner to ensure appropriate bone development and repair. (a) When mesenchymal progenitor (MP) cells express SOX9+, it indicates their commitment to the osteochondroprogenitor lineage differentiation. Indian hedgehog (IHH) binds to smoothened homologue (SMO), preventing the cleavage of GLI3 into the GLI3 repressor (GLI3R) while simultaneously activating the GLI2 activator (GLI2A), which subsequently leads to the expression of SOX9 and Runt-related transcription factor 2 (Runx2) in osteochondroprogenitor cells. (b) The Notch signaling pathway serves as a negative regulator of osteoblast differentiation. The binding of Notch to Jagged (JAG) or Delta-like proteins (DLLs) induces the proteolytic cleavage of Notch, allowing the Notch intracellular domain (NICD) to interact with RBPJ and Mastermind-like protein 1 (MAML1). This interaction subsequently affects downstream targets, including Hairy and Enhancer of Split (Hes) and Hes-related proteins with the YRPW motif (Hey), ultimately leading to the inhibition of osteoblast differentiation. (c) The canonical Wnt signaling pathway acts as a positive regulator of osteoblast differentiation. The binding of Wnt ligands to low-density lipoprotein receptor-related protein 5 (LRP5) or low-density lipoprotein receptor-related protein 6 (LRP6) and Frizzled (FZD) leads to the accumulation of β-catenin, facilitating its translocation to the nucleus. This translocation influences the expression of genes such as Runx2 and osterix (OSX), indicating that the cells begin to transition toward mature osteoblasts. (d) Bone morphogenetic protein (BMP) signaling pathway also serves as a positive regulator of osteoblast differentiation. The binding of BMP-2 or BMP4 to bone morphogenetic protein receptors (BMPR-I and BMPR-II) leads to the phosphorylation of Smad1, Smad5, or Smad8. Subsequently, these phosphorylated Smads form a complex with Smad4 and translocate to the nucleus, where they regulate gene expression, facilitating the transition of Runx2+OSX+ cells into mature osteoblasts. (e) Fibroblast growth factor (FGF) signaling pathway regulates the proliferation of preosteoblasts, osteoblast differentiation, and the function of mature osteoblasts. FGF binds to cell surface tyrosine kinase FGF receptors (FGFR1–FGFR4), triggering the activation of a cascade of signaling pathways, including MAPK, PI3K, STAT1, and protein kinase C (PKC). PS, presenilin; PTCH1, patched homologue 1. From a study by Salhotra et al. [9].

The osteogenic differentiation process of MSCs involves a sequence of transformations, including mesenchymal precursor cells, preosteoblasts, osteoblasts, and eventually mature bone cells. Precisely defining mesenchymal precursor cells poses a current challenge. Preosteoblasts encompass a diverse range of cells at various stages, each characterized by unique gene expressions, such as bone progenitor cells and immature osteoblasts. Mesenchymal precursor cells initiating the expression of the Sox9 gene are believed to possess the potential to differentiate further into chondrocytes or osteoblasts. The subsequent stage of cells transitions to expressing Runt-related transcription factor 2 (Runx2), followed by the expression of osterix (OSX), alkaline phosphatase (ALP), and type I collagen (Col1a1). The expressions of ALP and Col1a1 gradually intensify during the differentiation process into osteoblasts. Osteoblasts not only express the aforementioned genes but also synthesize bone matrix proteins, including osteocalcin (OCN), osteopontin (OPN), and bone sialoprotein (BSP). OCN expression is generally acknowledged as a marker for osteoblasts. Osteogenic differentiation can be simplistically categorized into three stages: the proliferation stage (involving insulin-like growth factor [IGF], transforming growth factor [TGF], fibroblast growth factor [FGF], etc., promoting the proliferation of mesenchymal precursor cells), the differentiation stage (where precursors gradually differentiate into osteoblasts under the influence of various growth factors), and the bone formation stage (during which secreted bone matrix proteins undergo mineralization and mature into bone tissue) [12, 13]. Sigmarsdottir et al. [12] further classified osteogenic differentiation into three metabolic stages by assessing concentrations of extracellular metabolites such as glucose, lactate, glutamine, glutamate, and ammonia during the process. The initial stage spans from day 1 to day 4, the second stage from day 5 to day 15, and the third stage from day 16 to day 28. This classification is based on variations in glycolysis and glutamine metabolism at different stages, accompanied by significant differences in mitochondrial activity during each stage [12]. The regulation of osteogenesis involves numerous and intricate signaling pathways, including, but not limited to, the Notch signaling pathway, mitogen-activated protein kinase (MAPK) signaling pathway, FGF signaling pathway, RhoA/ROCK signaling pathway, Wnt signaling pathway, BMP–Smad signaling pathway, and Hedgehog (HH) signaling pathway (Figure 1). Upon ligand–receptor interaction on the cell membrane, a specific pathway is activated, resulting in conformational changes in a series of upstream and downstream proteins under the influence of PKs. Ultimately, the signaling cascade is amplified, affecting gene expression through transcription factors. These various transcription factors exhibit not only upstream and downstream relationships but also form a complex regulatory network with feedback mechanisms among each other [14].

The subsequent section delves into several transcription factors, signaling molecules, and pathways that exert a substantial impact on osteogenic differentiation.

### 2.2. Key Transcription Factors in Osteogenic Differentiation

#### 2.2.1. Runx2

Runx2 stands out as a pivotal transcription factor crucial for osteogenesis. Its role extends to inducing the maturation and proliferation of chondrocytes during cartilage ossification. Furthermore, Runx2 plays a vital role in inhibiting the apoptosis of hypertrophic chondrocytes, steering their differentiation toward osteoprogenitor cells. This sets the stage for enhanced proliferation of osteoprogenitor cells and their subsequent differentiation into osteoblasts [15, 16]. The expression pattern of Runx2 reveals weak levels in undifferentiated MSCs, upregulation during the differentiation process into immature osteoblasts, reaching a peak, and subsequent downregulation in mature osteoblasts. Runx2 achieves this by directly regulating the expression of genes within Hh, FGF, Wnt, and parathyroid hormone-like hormone (Pthlh) signaling pathways. Additionally, Runx2 influences the homologous transcription factor distal-less homeobox 5 (Dlx5), further inducing the proliferation of MSCs and their differentiation into osteoblastic lineage cells [17]. By directly regulating FGF (FGR2 and FGFR3), Runx2 enhances the proliferation of osteoprogenitor cells [18]. In collaboration with Sp7 (OSX), Wnt, bone morphogenetic proteins (BMPs), and IGF-1, Runx2 facilitates the differentiation and maturation of osteoprogenitor cells. It induces the expression of major bone matrix protein genes, such as Col1a1, Spp1, Ibsp, Bglap2, and Fn1. The expression and activity of Runx2 are intricately influenced by Hh, FGF, Wnt, Pthlh, and Sp7. Additionally, Runx2 contributes to bone resorption regulation by influencing Tnfsf11 [19]. Wnt, BMP, and Notch signaling pathways orchestrate the regulation of Runx2 expression. The absence of Runx2 is implicated in deficient human skull development, manifesting as craniosynostosis and incomplete closure of fontanelles and sutures, coupled with underdeveloped clavicles [20].

#### 2.2.2. OSX

OSX, also known as Sp7, represents a bone cell-specific transcription factor belonging to the SP/KLF family [21]. Serving as a downstream protein of Runx2, OSX exhibits specific expression in osteoblasts during both endochondral and intramembranous ossifications, demonstrating low expression levels in prehypertrophic chondrocytes and complete absence in hypertrophic chondrocytes [22, 23]. Its involvement in osteogenic differentiation is marked by the regulation of key genes such as BSP, OCN, OPN, fibromodulin, osteonectin, and Col1a1 [24–26]. OSX, in addition to being influenced by signaling pathways regulating Runx2, can be independently regulated by BMP-2 and the Wnt pathway [27, 28]. The significance of OSX is underscored by its critical role in embryonic skeletal development; embryos lacking the OSX gene exhibit a deficiency in osteoblast differentiation. Notably, the absence of OSX or its mutations leads to bone defects and delayed osteogenic differentiation in postnatal animals [29–31].

#### 2.2.3. DLX5

DLX5 belongs to the DLX homeobox family of genes. During embryonic development, DLX5 is expressed in cells of various tissues, including the branchial arches, brain, and skeleton [32, 33]. The overexpression of DLX5 may lead to excessive bone growth through the PI3K/Akt signaling pathway and can also promote hypertrophy and apoptosis of MSCs derived from human cartilage [34, 35]. Studies indicate that DLX5, under mechanical stimulation, influences osteogenic differentiation through the Notch signaling pathway [36–39]. Simultaneously, its expression is upregulated in the BMP-2-mediated osteogenic differentiation process. Deletion of DLX5 in mice results in postnatal death and craniofacial abnormalities, such as malformed mandibles [33].

#### 2.2.4. Activating Transcription Factor 4 (ATF4)

ATF4 is a mammalian DNA-binding protein expressed widely in various cell lineages. ATF4 plays a crucial role in cell differentiation, proliferation, apoptosis, and redox reactions, contributing significantly to maintaining skeletal homeostasis. ATF4 promotes the differentiation of MSCs into the osteoblastic lineage, increases osteoblast activity, and inhibits OC formation simultaneously [40–42]. Acting as a downstream factor of Runx2, ATF4 upregulates the expression of OCN and OSX through interaction with Runx2 [43, 44]. ATF4 deficiency affects the differentiation of mesenchymal cells into osteoprogenitor cells but does not impact mesenchymal cell proliferation [45]. Research suggests that endoplasmic reticulum stress assists osteoblast differentiation through the activation of the PERK-eIF2α-ATF4 pathway [46]. Besides the PERK-eIF2α pathway, ATF4 expression is also regulated by pathways such as BMP. BMP-2, an upstream factor, can influence ATF4′s function through various mechanisms [47]. ATF4 plays a crucial role in skeletal development, maintaining skeletal homeostasis, and bone remodeling. Loss of ATF4 leads to decreased bone strength, deterioration of bone mass, and bone structure. ATF4-deficient mice exhibit reduced femoral bending strength and fracture resistance [45, 47].

### 2.3. Key Signaling Pathways in Osteogenic Differentiation

#### 2.3.1. Wnt/β-Catenin Signaling Pathway

The Wnt/β-catenin signaling pathway plays a crucial role in regulating embryonic development, stem cell renewal, and tissue health. It is one of the primary regulatory signaling pathways in the process of osteogenic differentiation. Wnt is a secreted protein, with Wnt3a, Wnt5a, and Wnt1 acting as extracellular signals that, upon binding to the Frizzled protein, activate the Wnt/β-catenin signaling pathway. β-Catenin is the central mediator of this pathway, and signal transduction maintains homeostasis by precisely controlling the level of β-catenin in the cytoplasm. The major components of the Wnt/β-catenin signaling pathway include membrane segments (FZD, low-density lipoprotein receptor-related protein 5 [LRP5], and low-density lipoprotein receptor-related protein 6 [LRP6]), cytoplasmic segments (DvL, AXIN, APC, glycogen synthase kinase-3β, casein kinase I, and β-catenin), and nuclear segments (T-cell factor [TCF]/lymphoid enhancer factor [LEF], β-catenin, and downstream genes) [48]. The Wnt signaling pathway can be divided into the classical pathway and the nonclassical pathway. The classical Wnt pathway, also known as the Wnt/β-catenin signaling pathway, involves the stabilization and accumulation of β-catenin in the cytoplasm, followed by translocation to the nucleus to activate target genes through TCF/LEF transcription factors. The nonclassical pathways include the Wnt/calcium signaling pathway and the Wnt/planar cell polarity (PCP) signaling pathway. The classical Wnt pathway primarily controls cell proliferation, while the nonclassical Wnt pathways regulate cell polarity and migration. These two major pathways can interactively regulate each other. The activation of the Wnt signaling pathway is associated with various proteins, such as Wnt-10b and Wnt-3a. Proteins like Dickkopf (such as Dkk1 and Dkk2) and sclerostin (Sost) bind to the extracellular structures of LRP5 and LRP6, interfering with the action of Wnt proteins and thereby inhibiting the signaling transduction of this pathway. The Wnt signaling pathway interacts with other signaling pathways. Among them, the PI3K/Akt/mTOR, HH, and other pathways have inhibitory effects on it, while the TGF-β pathway has a promoting effect. The BMP and Notch pathways may exert inhibitory or promoting effects depending on the context [48–53]. The Wnt/β-catenin signaling pathway promotes the differentiation of preosteoblasts into osteoblasts and facilitates the maturation of osteoblasts. It also plays a crucial role in regulating bone metabolism in normal bone tissue. For instance, the inactivation of LRP5 leads to osteoporosis-pseudoglioma syndrome (OPPG) [54]. Loss or activation of LRP5 results in decreased or increased bone mass, as confirmed in mouse models [55]. β-Catenin is equally crucial, as the ablation of β-catenin in mesenchymal progenitor cells prevents them from differentiating into osteoblasts [56]. It is now known that β-catenin, together with TCF-1, can directly stimulate the transcription of Runx2 [57].

#### 2.3.2. Notch Signaling Pathway

The Notch pathway plays a crucial role in skeletal development and metabolism. Currently, four transmembrane receptors have been identified in the Notch pathway, and these receptors consist of extracellular domain (NECD), transmembrane domain, and intracellular domain (NICD) [58]. Notch ligands are also transmembrane proteins and can be classified into classical ligands and nonclassical ligands. Classical ligands involve direct intercellular signaling, where a membrane-bound ligand on one cell binds to the Notch receptor expressed on another cell, activating the pathway. Five human Notch receptors have been identified: Delta-like (Dll)-1/3/4 and Jagged1/2. Ligands and receptors from different species can freely combine and bind to each other [59]. After ligand–receptor binding, their cleavage site is exposed for cleavage by integrins and a disintegrin and metalloproteinase (ADAM). Following the second cleavage by the γ-secretase complex, NICD is released into the cytoplasm. NICD undergoes nuclear translocation and, in conjunction with mastermind-like 1-3 (MAML1-3), recombining binding protein suppressor of hairless (RBP-Jκ), and coactivators, which collectively activates the expression of target genes [58, 59]. The typical target genes of the Notch signaling pathway include enhancers for hairy and enhancer of split (Hes) family members, such as Hes1, Hes5, Hes6, and Hes7, as well as those associated with the YRPW motif (Hey) family, including Hey1, Hey2, and Hey-like (HeyL). The noncanonical Notch signaling pathway refers to the interaction and binding of NICD with intracellular proteins other than Rbpjκ and MAML. Notch1 inhibits osteoblast differentiation and Wnt/β-catenin signal transduction. Overexpression of Notch reduces the cytoplasmic levels of β-catenin and the stimulation of ALP activity by Wnt3 [60]. However, studies have suggested that the activation of Notch1 in osteoblasts inhibits Wnt antagonists, such as Sost and Dickkopf 1 (Dkk1). In other words, Notch can enhance Wnt/β-catenin signaling transduction [61]. Although the results are contradictory, the direct inhibitory effect of Notch on Wnt signal transduction predominates [60, 62]. Lack of Notch1 and Notch2 in preosteoblasts increases the number of osteoblasts and bone formation [63]. Hes and Hey proteins inhibit the function of Runx2, suppressing osteoblastogenesis [64]. Mutations in the Notch1 and Notch2 genes may lead to brachydactyly and Hajdu–Cheney syndrome (HCS), respectively.

#### 2.3.3. FGF Pathway

FGF pathway is a crucial signaling network involved in various biological processes, including cell proliferation, differentiation, migration, and survival. This pathway comprises multiple members of the FGF family, their receptors, and downstream signaling molecules, with FGF-2 and FGF18 being particularly significant in skeletal development. FGF-2, also known as basic FGF (bFGF), primarily exerts its biological functions by binding to FGFR1, FGFR2, and FGFR3. Different doses of FGF-2 can induce osteogenesis in vitro [65, 66]. The influence of FGF-2 on osteogenesis may be limited to the early stages of supporting cell proliferation, lineage specification, and differentiation. Mice with FGF-2 knockout exhibit reduced bone mass and increased marrow adiposity, while exogenous FGF-2 can inhibit adipogenesis and restore some ALP activity and bone nodule formation in in vitro experiments [67, 68]. FGF-18 is a member of the FGF 8 subfamily, capable of binding to FGF receptors (FGFR1-4). It primarily activates four intracellular signaling pathways: RAS-MAPK, PI3K-Akt, PLC*γ*, and STAT. FGF-18 is mainly involved in cell proliferation [69, 70]. FGF-18 regulates the development of chondrocytes, promoting chondrocyte proliferation and differentiation into hypertrophic chondrocytes during early mouse embryonic development. However, it later switches to negative regulation [71]. FGF-18 enhances PI3K/ERK and Smad1/5/8 pathways by blocking noggin (a BMP-2 antagonist), influencing bone regeneration [72]. Mice lacking FGF-18 exhibit reduced ossification in the cranial suture region, along with developmental or morphological abnormalities in the skull, mandible, ribs, radius, tibia, and other bones [73].

#### 2.3.4. HH Signaling Pathway

HH belongs to the family of paracrine signaling factors and has three paralogs: sonic HH (SHH), Indian HH (IHH), and desert HH (DHH). The main receptor for HH is the transmembrane protein Patched (PTCH). The signaling pathway of HH is unique: in the absence of ligand, PTCH inhibits the activity of another transmembrane protein, Smoothened (SMO), thereby suppressing signal transduction. When SMO is inactive, the fusion inhibitory factor (SUFU) and protein kinase A (PKA) promote the cleavage of GLI proteins into repressor forms (GLI-R), which enter the cell nucleus and inhibit the transcription of target genes. Conversely, when the HH ligand is present, this series of inhibitory effects is blocked, allowing GLI proteins to enter the cell nucleus and activate the transcription of relevant target genes [74, 75]. SHH is expressed by mesenchymal progenitor cells during the limb bud patterning process, which involves cell proliferation, differentiation, and migration, ultimately leading to the formation of the morphology and function of the limbs. The absence of the SHH gene results in the loss of cranial bones, vertebrae, ribs, and even distal limb structures, as well as conditions such as cyclopia [76]. Additionally, SHH induces an aging-related secretory phenotype and apoptosis of chondrocytes in human osteoarthritis MSCs (OA-MSCs) [77]. DHH, on the other hand, plays a critical role in regulating spermatogenesis in mammals, and a deficiency in DHH can lead to human gonadal dysgenesis [78, 79]. IHH is the only member of the HH family expressed in chondrocytes during endochondral ossification in the process of intramembranous ossification. It is synthesized by hypertrophic chondrocytes and participates in chondrocyte hypertrophy. IHH plays a crucial role in the differentiation of MSCs into osteoprogenitor cells and the proliferation of osteoblasts [75, 80]. Mice lacking IHH are unable to form osteoprogenitor cells, and intramembranous ossification in the cranial vault fails to produce osteoblast differentiation [81].

#### 2.3.5. BMP Pathway

BMPs belong to the TGF-beta (TGF-β) superfamily and are multifunctional secreted signaling molecules. Over 20 BMP proteins have been identified, with BMP-2 and BMP-4 playing representative roles in osteoblast differentiation. BMP proteins primarily act through the classical Smad signaling pathway and the nonclassical MAPK signaling pathway (or Smad-dependent and Smad-independent groups). The receptors for BMP are serine/threonine kinase receptors, categorized as type I (BMPR-I) and type II (BMPR-II). BMP forms a complex with both receptors, leading to phosphorylation of BMPR-II, followed by phosphorylation of BMPR-I, and subsequently phosphorylation of Smad1, Smad5, and Smad8. Phosphorylated Smads form a complex with Smad4, translocate to the cell nucleus, and activate the transcription of target genes such as Runx2 and OSX. The MAPK pathway includes p38, ERK, and JNK. In the process of embryonic development, BMP plays a crucial role in the formation of the mesoderm and the regulation of dorsal–ventral patterning in the ectoderm [82]. BMP-2, BMP-4, BMP-5, BMP-6, and BMP-7 are expressed in distinct regions during embryonic cartilage development. BMP-2 and BMP-4 recruit MSCs into cartilage, while BMP-6 functions in the differentiation of terminal cartilage. Additionally, BMP-2, BMP-9, and BMP-7 have been found to induce cartilage and bone formation in vitro [82, 83]. The Smad signaling pathway upregulates the expression of Runx2, subsequently activates downstream gene transcription, and influences osteogenic differentiation [84]. Simultaneously, this pathway involves the Msx2 mechanism, activating the expression of OSX [28]. It is worth mentioning that BMP-3 interferes with BMP signal transduction [85]. Mice with BMP-2 mutations die around 7–10 days of embryonic development with accompanying heart defects. Mice with BMP-4 mutations die around 6–9 days of embryonic development with defects in mesoderm formation [86, 87]. Interestingly, although BMP is involved in skeletal development and formation, mice with BMP-2/4 knockout do not exhibit obvious skeletal diseases [88]. Mice with BMP-5 gene mutations show reduced external ear size and sternal and rib abnormalities. Mice with BMP-6 gene mutations experience delayed sternal ossification. Mice with BMP-7 deficiencies exhibit abnormalities in ribs, hind limbs, and cranial bones [89–91]. This may result from crosstalk between the BMP signaling pathway and other signaling pathways. For instance, there are various interaction modes and mutual influences between the Wnt signaling pathway and the BMP signaling pathway [92]. The classical Wnt signaling pathway participates in BMP-9-induced MSC osteogenic differentiation [93]. The Smad signaling pathway triggered by BMP-2 enhances the activity of β-catenin, accelerates the phosphorylation of β-catenin, and increases the concentration of β-catenin [94].

The interplay between signaling pathways is complex, exemplified by the well-studied crosstalk between the Wnt and BMP pathways, which occurs at multiple stages of molecular transduction, including Runx2, β-catenin, Dsh, and GSK3 [95]. In simple terms, BMP enhances the osteogenic differentiation of MSCs, but the proliferative effects induced by the canonical Wnt pathway are counteracted. Wnt signaling may make undifferentiated MSCs more responsive to BMP [96]. The Notch pathway, considered a negative regulator of osteogenesis, reduces β-catenin levels upon overexpression, thereby inhibiting the Wnt pathway. BMP-2 may activate the Notch target gene Hey1, which in turn suppresses Runx2′s transcriptional activity on osteogenic genes [97]. However, Notch signaling activation in MC3T3-E1 cells overexpressing NICD can stimulate BMP-2-induced osteoblast (IOB) generation [98]. The HH pathway primarily influences osteogenic differentiation through Runx2, working in concert with the BMP pathway to promote osteogenesis [99], while also exhibiting some interaction with the Wnt pathway. Notably, although IHH signaling is independent of β-catenin, the absence of β-catenin hampers the downstream osteogenic differentiation induced by IHH signaling [100]. Similarly, FGF-2 partially regulates osteogenic differentiation through Runx2, modulating the expression of endogenous BMP-2 to collaboratively promote osteoblast differentiation in the skull [101]. However, FGF may inhibit β-catenin from binding to TCF/LEF, thereby suppressing classic Wnt-induced osteogenesis [102]. Signaling pathways achieve mutual regulation by acting on the receptors or target genes of other pathways, and differing targets may yield opposing effects.

### 2.4. Differences in Osteogenic Differentiation Between MSCs From Different Sources

MSCs derived from distinct sources exhibit variations in gene expression, demonstrating diverse trends in differentiation and functionality [103, 104]. Notably, BM-MSCs, AT-MSCs, and dermal-derived MSCs demonstrate ALP activity even in the absence of osteogenic induction medium. In this context, BM-MSCs outperform AT-MSCs, while dermal MSCs exhibit relatively low expression, and cardiac MSCs fail to express ALP activity. Introduction of osteogenic induction medium induces a morphological transition in BM-MSCs from spindle-shaped to polygonal within 3–4 days. Subsequently, positive ALP staining is evident around day 7, showing a cumulative trend by day 14. Alizarin red staining reveals the presence of calcium nodules, initially in small amounts at day 14, becoming more pronounced by day 21. Although the latter time point serves as a common assessment node in most studies, achieving consensus on the peak time remains challenging. In line with prior research, the observation of calcium deposits in the extracellular matrix after 7 days of induction and mineralization nodes after 21 days in jaw bone-derived MSCs corroborates previous findings [105]. Comparative analyses of osteogenic differentiation abilities indicate the following ranking: BM-MSCs > AT-MSCs > PDLSCs > DFSCs > DPSCs > UC-MSCs ≥ GMSCs [103, 104, 106–109]. Furthermore, SHEDs display a stronger osteogenic capacity than DPSCs but a weaker one than PDLSCs [110]. Notably, SCAPs exhibit superior osteogenic ability compared to DPSCs. Quantitative assessments encompassing ALP staining, alizarin red staining, and gene expression analysis of ALP, COL1A1, Runx2, and SPP1 supported these findings. It is noteworthy that while BM-MSCs and AT-MSCs display a low proliferative capacity, UC-derived MSCs exhibit the highest proliferation efficiency [106], suggesting a potential decoupling of differentiation and proliferative activity. However, a study by Davies et al. [111] challenges these observations, reporting that DPSCs produce significantly more minerals than AT-MSCs and BM-MSCs [111]. In a comparison by Li et al. [112] assessing the osteogenic ability of BM-MSCs, AT-MSCs, WJ-MSCs, and PL-MSCs, it was found that WJ-MSCs and PL-MSCs formed a greater number of bone nodules than BM-MSCs and AT-MSCs [112]. Importantly, these discrepancies in osteogenic potential may be attributed to donor age, as underscored in prior investigations [106, 113, 114].

## 3. Effect of Different Cells on Osteogenic Differentiation of MSCs

### 3.1. Macrophages

Macrophages assume a pivotal role in the local healing and regeneration of bone subsequent to a fracture [115]. Their recruitment to the wound site is facilitated by proinflammatory chemokines, including interleukins (IL-1, IL-6, etc.), tumor necrosis factor-α (TNF-α), macrophage colony-stimulating factor (M-CSF), and inducible nitric oxide synthase (iNOS). Upon arrival at the site, macrophages coordinate both acute and chronic inflammatory responses, thereby actively contributing to the intricate process of bone repair [116, 117]. Macrophages undergo an initial transition into the M1 phenotype, characterized by proinflammatory effects. M1 macrophages exhibit the secretion of cytokines such as TNF-α, IL-1, IL-6, and chemokines including C-X-C motif chemokine ligand 2, 8, and 10 (CXCL2, CXCL8, and CXCL10) [118, 119]. They play a critical role in the defense against bacteria at the wound site. The M1 phenotype serves as a precursor to OCs and has the capacity to activate OCs. However, prolonged activation of M1 macrophages may lead to bone metabolic diseases, attributed to heightened bone resorption, and could contribute to other inflammatory conditions [10, 120]. Striking a balance in the duration of M1 activation is critical for optimal bone healing and preventing adverse inflammatory outcomes. As the levels of proinflammatory cytokines decrease and anti-inflammatory cytokines increase, the M1 phenotype transitions to the M2 phenotype. The M2 phenotype functions as anti-inflammatory macrophages, releasing cytokines such as IL-10, IL-4, TGF-β, and vascular endothelial growth factor (VEGF). Importantly, M2 macrophages exhibit an inhibitory effect on OC formation [116, 121]. Concurrently, during this phase, MSCs undergo differentiation into preosteoblasts, contributing to the subsequent stages of bone regeneration and repair. This orchestrated shift from proinflammatory to anti-inflammatory macrophage phenotypes is a crucial aspect of the finely tuned regulatory mechanisms governing bone healing processes.

Contemporary research consistently demonstrates that the M2 phenotype can positively influence the osteogenic differentiation of MSCs when cocultured using various modes [122–124]. This enhancement is primarily mediated by a diverse array of cytokines, including soluble factors such as oncostatin M (OSM), BMP-2, BMP-4, TGF-β1, CCL17, and CCL22 (chemokines) [125, 126]. The underlying mechanisms predominantly involve the OSM and BMP signaling pathways. Additionally, IL-10 mRNA released within exosomes contributes to the facilitation of this process [119]. Macrophages exert a positive influence on the osteogenesis of MSCs by downregulating reactive oxygen species (ROS) [124]. Nevertheless, the impact of macrophages on the osteogenic differentiation of MSCs remains a subject of debate. Zhanget al. [122] suggested that the M1 phenotype might enhance osteogenesis but predominantly in the early stages of osteogenic differentiation [122]. In contrast, Chen et al. [127] argued that the conditioned medium from macrophages had an inhibitory effect on BMP-2-induced osteogenic differentiation of human mesenchymal stem cells (hMSCs) compared to the conditioned medium from undifferentiated monocytes [127]. In the realm of molecular mechanisms, M1 macrophages exert their influence on osteogenic differentiation primarily through the secretion of TNF-α and IL-6. The role of TNF-α in osteogenic differentiation varies across different stages. Research suggests that in the early stages of osteogenic differentiation, TNF-α can upregulate the expression of key factors such as BMP-2, OSX, Runx2, OCN, and activate the Wnt pathway. This effect is mainly mediated through the NF-κB pathway, thereby promoting osteogenic differentiation. However, in the presence of preosteoblasts, TNF-α may inhibit osteogenic differentiation by activating Smad ubiquitination regulatory factor-1 and Smad ubiquitination regulatory factor-2 (SMURF1 and SMURF2), leading to Smad inhibition [128–131]. Additionally, the impact of TNF-α is contingent on its concentration and duration of action [132]. On the other hand, IL-6 inhibits osteogenic differentiation through the MEK2/ERK and PI3K/Akt2 pathways, while it can have a promoting effect via the STAT3 pathway [133]. Xiang et al. [134] proposed a potential relationship between β-catenin and IL-6, suggesting that IL-6 may influence osteogenic differentiation by inhibiting the classical Wnt signaling pathway or inducing the nonclassical Wnt signaling pathway [134]. In a study by Tu et al. [135], it was demonstrated that IL-23-secreting macrophages activate the STAT3 and β-catenin pathways, thereby directly inducing osteogenesis in MSCs. Inhibition of the IL-23 signaling pathway resulted in reduced mRNA expression of key osteogenic markers, including Runx2, ALP, and OCN [135]. Tang et al. [136] observed that the osteogenic differentiation process was hindered by noninflammatory factors when macrophage phenotypes were cocultured with AT-MSCs on three-dimensional (3D) PLGA/PCL scaffolds [136]. Similarly, in a scaffoldless 3D culture model, they concluded that macrophages exhibited an inhibitory effect on osteogenic differentiation when cocultured with AT-MSCs in spheroids, with the M1 phenotype showing a stronger inhibitory effect [137]. It is noteworthy that these outcomes may be influenced by variations in 3D culture systems and/or serum composition. The culture mode and cell proportion can profoundly influence the outcomes of cell differentiation. Luo et al. [124] posited that the direct cocultivation of macrophages and MSCs yields more favorable conditions for the osteogenic differentiation of MSCs when compared to indirect cocultivation or the utilization of conditioned medium [124]. Moreover, Romero-Lopez et al. [123] proposed that an optimal cocultivation effect is achievable by maintaining a macrophage-to-hMSC ratio of 5:1 [123]. In summary, macrophages play a pivotal role in the natural bone repair process, exhibiting a positive impact on bone formation in two-dimensional (2D) in vitro experiments. Nevertheless, the precise mechanisms underlying the observed inhibitory effects in the 3D culture system necessitate further elucidation. Additionally, the intricacies of signal transduction during direct contact culture between macrophages and MSCs warrant thorough exploration.

### 3.2. OCs

**Figure 2:**
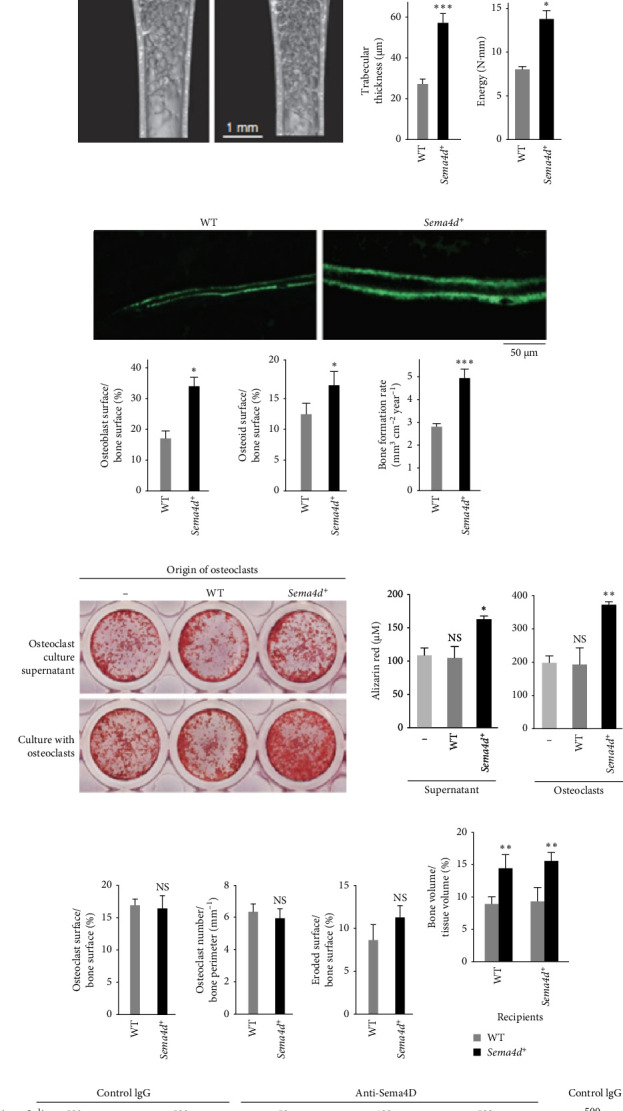
Inhibition of bone formation by osteoclast-derived Sema4D. (a) Microcomputed tomography (µCT) of the proximal femur of the wild-type (WT) and Sema4d^−/−^ mice (top left, axial view of the metaphyseal region; bottom left, longitudinal view). Bone volume and trabecular thickness were determined by µCT analysis (middle). Maximum load to failure and energy resorption were determined by the three-point bending test (right). (b) Bone formation, as observed by calcein double labeling at an interval of 4 days (top), and the parameters for osteoblastic bone formation, as determined by bone morphometric analysis (bottom). (c) Effect of osteoclast culture supernatant or coculture with osteoclasts on bone nodule formation. Left, alizarin red staining; right, amount of the alizarin red. (d) The parameters for osteoclastic bone resorption, as determined by bone morphometric analysis. (e) Bone volume after adoptive transfer of WT or Sema4d^−/−^ bone marrow (BM) cells to WT (left) and Sema4d^−/−^ (right) mice. (f) Effect of antibody to Sema4D (anti-Sema4D) on bone formation in WT osteoblasts cocultured with WT or Sema4d^−/−^ osteoclasts, alizarin red staining. *⁣*^*∗*^*p* < 0.05; *⁣*^*∗∗*^*p* < 0.01; *⁣*^*∗∗∗*^*p* < 0.005; ND, not detected; NS, not significant. Error bars show SEM From a study by Negishi-Koga et al. [146].

OCs, originating from macrophages or hematopoietic stem cells, function in bone resorption during the process of bone remodeling. In contrast, osteoblasts, derived from MSCs, are responsible for bone formation. The intricate and dynamic interplay between these cell types is essential for maintaining bone health. Communication between OCs and osteoblasts encompasses various modes, including the expression of cell membrane surface factors, secretion of influencing factors, and the release of extracellular vesicles (EVs) [138]. This complex cascade of interactions is vital for the delicate balance between bone resorption and formation in the maintenance of overall bone homeostasis. Among the osteoblast-derived factors, Ephrin B2, Fas ligand, semaphorin 3A (Sema3A), WNT16, and osteoprotegerin have been identified as inhibitors of OC proliferation and differentiation. On the other hand, M-CSF, receptor activator of NF-κB ligand (RANKL), and WNT5A are known to promote OC proliferation and differentiation. Turning the focus to OCs, studies have investigated the impact of conditioned medium from OCs, including OC precursors (pOCs) and mature OCs (mOCs), on the osteogenic differentiation of MSCs. The findings indicate that BMP-2, BMP-4, BMP-6, BMP-7 proteins, Wnt10b, IGF-1, sphingosine 1-phosphate (S1P), platelet-derived growth factor-BB (PDGF-BB), IGF binding protein 5 (IGFBP5), and CXCL12 play pivotal roles in promoting osteoblast differentiation and migration. In specific terms, OCs exhibit the ability to recruit bone progenitor cells to bone remodeling sites through the secretion of S1P and BMP-6. They further stimulate bone formation by activating the Wnt and BMP signaling pathways. Conversely, factors such as semaphorin 4D (Sema4D), leukemia inhibitory factor, Sost, and exosomal miR-214-3p play inhibitory roles in this context [139–144]. The expression of Sema4D in OCs inhibits bone formation in osteoblasts, being present in OCs but lacking in osteoblasts. Sema4D exists in two forms in vivo: as a type I membrane protein and as soluble Sema4D (sSema4D). The latter is cleaved from the OC membrane and released in response to RANKL stimulation, and it exhibits functional activity in inhibiting osteoblastogenesis [145]. When the Sema4D gene expression is deficient in OCs in mice, there is a significant increase in bone volume and bone mass in these mice. In in vitro osteogenic differentiation experiments, coculturing Sema4D-deficient OCs with osteoblasts results in higher expression of osteogenic-related genes such as Col1a1 in osteoblasts compared to the control group, and more bone nodules are formed. At the same time, the deficiency of the Sema4D gene does not affect the quantity of OCs and osteoblasts. The research also indicates that Sema4D suppresses factors produced by OCs that promote bone formation. Sema4D primarily binds to the receptor Plexin-B1 on osteoblasts, inhibiting bone formation by suppressing IGF-1 signaling and modulating osteoblast movement. IGF-1, in turn, induces the osteogenic differentiation of MSCs through the IRS-PI3K-Akt-mTOR pathway. The absence of Plexin-B1 also favors bone formation. Additionally, the effects of Sema4D are partially mediated by other receptors, such as Plexin-B2 [146, 147] (Figure 2). Sema4D may regulate bone formation through additional mechanisms [148]. A. Deb Roy and colleagues discovered that OCs can induce contact repulsion in osteoblasts (where OCs and osteoblasts briefly interact and then separate). They developed the first optogenetic tool capable of precisely controlling the activation of Plexin-B1 by light in both location and timing, confirming that this effect is coordinated through the RhoA-ROCK-mediated redistribution of β-Pix via Plexin-B1 [149]. Additionally, Sema4D-mediated bone resorption may be linked to ovarian function. In a study by Dacquin et al. [150], female mice with Sema4D deficiency exhibited increased bone mass phenotype after sexual maturity, which was eliminated by ovariectomy. Moreover, Sema4D-deficient mice showed reproductive impairment, suggesting that Sema4D is a factor that links bone remodeling and ovarian function [150]. The inhibitory effect of LIF on OCs may involve its stimulation of prostaglandin synthesis by osteoblasts. Furthermore, osteoprotegerin competitively inhibits the coreceptor LRP5 in the Wnt signaling pathway, thereby suppressing the pathway and influencing bone formation. Additionally, it is noteworthy that ABs derived from OCs have a positive effect on the osteogenesis of MSCs. These ABs can be derived from both pOC and mOC. pOC-ABs are rich in PDGF-BB, which is particularly conducive to promoting angiogenesis. Conversely, MOC-ABs are rich in RANK, promoting osteogenesis through the reverse signal of receptor activator of RANKL [151–153]. Macrophages and OCs play crucial roles in maintaining bone homeostasis, and the cytokines they secrete serve as guidance cues for MSCs homing, proliferation, and differentiation. Despite their pivotal role, there is a paucity of direct coculture experiments studying their specific effects on the osteogenic differentiation of MSCs. The challenges in culturing and differentiating OCs in vitro, coupled with potential impacts of osteogenic induction medium on cells, present significant obstacles in the exploration of cell coculture dynamics. Overcoming these challenges is essential for a comprehensive understanding of the intricate interplay between macrophages, OCs, and MSCs in the context of bone tissue homeostasis. Further research and optimization of coculture methodologies are needed to unveil the full spectrum of interactions among these key players in bone health.

### 3.3. ECs

The vasculature indeed plays a crucial role in providing essential nutritional supplies for the repair processes in tissues and serves as pathways for cell homing. In bone tissue engineering, the challenge of internal cell death due to insufficient oxygen and nutrient supply is a significant concern. One strategy to address this issue involves the addition of ECs to microtissues to achieve prevascularization. However, several challenges remain in this approach. Achieving the orderly arrangement of the formed vascular structure, ensuring practical integration into the host vasculature, and addressing potential immunogenicity issues are areas that require improvement. In efforts to promote osteogenic differentiation, coculture experiments have employed ECs from various sources, showcasing the versatility of this approach. The sources include endothelial progenitor cells (EPCs) [154–157], human umbilical vein endothelial cells (HUVECs) [158, 159], and human microvascular endothelial cells (HMECs) [160, 161]. ECs play a pivotal role in promoting osteogenesis through two primary mechanisms. Firstly, there is an indirect promotion involving the establishment of vascular channels to facilitate nutrient delivery, cocultivation with MSCs to sustain MSC stemness and viability, and the secretion of cytokines and EVs to stimulate the migration and proliferation of osteoblasts. Notable factors released include BMPs (BMP-1, BMP-3, BMP-6, BMP-7, and BMP-8), VEGF, and TGF-β. Secondly, MSCs contribute to osteogenic differentiation by secreting growth factors such as BMP-2, endothelin-1 (ET-1), and IGF. This is evidenced by the upregulation of key markers including ALP, BMP-2, osteonectin, and OPN. Xu et al. [57] utilized quantitative real-time polymerase chain reaction (qRT-PCR) and gene knockout techniques to demonstrate that the activation of the p38 MAPK pathway emerges as a crucial factor in augmenting the osteogenic differentiation of MSCs through the indirect cultivation of EPCs [57]. In a separate investigation by Petrillo et al. [160], it was observed that ECs heightened the glycolytic metabolism of stem cells from SHED while concurrently diminishing their reliance on mitochondrial aerobic metabolism. This metabolic shift allowed MSCs to preserve their osteogenic differentiation capacity even in hypoxic conditions [160, 162]. Notably, when ECs and MSCs were cocultured at a 1:1 ratio, optimal ALP activity and matrix mineralization were achieved in a 2D environment. Moreover, the application of cyclic tensile stress within the coculture setting has been demonstrated to enhance the expression of Runx2 and Col-1 genes, along with an increase in ALP activity in MSCs [158, 163, 164]. Concurrently, ECs exhibit the capacity to stimulate osteoblasts, leading to the secretion of various factors including VEGF, angiopoietin-1 (ANG-1), hepatocyte growth factor (HGF), TGF-β, and PDGF-beta (PDGF-β). This, in turn, facilitates the recruitment and migration of ECs, promoting angiogenesis. This reciprocal interaction establishes a beneficial cycle fostering both osteogenesis and angiogenesis. To date, no study has demonstrated a direct promotional effect of EPC-EVs on osteogenic differentiation. The influence of direct cell-to-cell contact, mediated by factors like integrin, on osteogenic differentiation remains an area that requires further exploration, given the tight adhesion observed during coculture.

### 3.4. Nerve Cells

Recent studies have illuminated the critical role of the nervous system in governing the processes of bone and vascular development, regeneration, and remodeling [165]. Sensory and sympathetic nerves exhibit widespread distribution within bone tissues, including the periosteum, BM cavity, and even the Haversian and Volkmann's canals of cortical bone. This extensive innervation plays a pivotal role in regulating osteogenesis and maintaining bone homeostasis [166]. The intricate relationship between the nervous system and bone repair after trauma is highlighted by the dynamic rearrangement of blood vessels and nerves. Notably, during the early stages of bone repair, sprouting nerve fibers are observed in the chondrogenic area prior to ossification, coinciding with the appearance of blood vessels. This simultaneous involvement of nerve fibers and blood vessels suggests a coordinated and interdependent process in response to bone injury. The recruitment of nerve cells to injured areas during bone repair plays a crucial role in facilitating bone regeneration and angiogenesis [167]. Nerve cells release angiogenic and osteogenic factors, contributing to the orchestration of a regenerative microenvironment. Neurotransmitters or neuropeptides released by nerves exert their effects by binding to target cell receptors. In the context of bone tissue regulation, sensory nerves release calcitonin gene-related peptide (CGRP) and substance P (SP), two neuropeptides that play a positive regulatory role in bone physiology. CGRP, for instance, contributes to the formation of cyclic adenosine monophosphate (cAMP) in osteoblasts. This, in turn, stimulates the proliferation and differentiation of bone precursor cells, upregulates the expression of osteogenic genes, and enhances the overall activity of osteoblasts. Furthermore, CGRP binds to calcitonin receptors, reducing the activity of OCs, thereby promoting bone formation. The bidirectional communication between bone tissue and nerves is highlighted by the feedback mechanism wherein bone tissue secretes neurotrophic factors. This feedback mechanism serves to modulate the number and type of nerves required for optimal bone homeostasis and regeneration. Neurotrophic factors play a crucial role in guiding bone innervation by binding to functional receptors known as transmembrane tyrosine kinases (RTKs). This interaction leads to the upregulation of osteoblast activity, promoting both osteogenesis and vascularization [168]. The intricate connection between the nervous system and bone physiology is further highlighted by neural guidance molecules that regulate axon growth and participate in processes such as angiogenesis and bone regeneration through various signaling pathways. A significant member of this family of molecules is Sema3A, a protein that acts on both osteoblasts and OCs. Sema3A exhibits the ability to inhibit axon growth and cell migration in an autocrine manner, playing a crucial role in regulating the development of sensory nerves. Indirectly, Sema3A promotes the differentiation of osteoblasts, inhibits the differentiation of OCs, and contributes to the regulation of bone remodeling. The loss of Sema3A has been associated with severe bone formation disorders and disarrangements, emphasizing its essential role in maintaining bone homeostasis [169]. Currently, research involving nerve cells related to osteogenic differentiation is limited, and various cell lines are employed to study sympathetic and sensory nerve interactions in this context. Sympathetic nerve-related cell lines include the rat pheochromocytoma cell line PC12 [170], superior cervical ganglion (SCG) cells [171], and human neuroblastoma cells (SH-SY5Y) [172]. On the other hand, sensory nerve cells commonly studied include dorsal root ganglion neurons (DRGs) and trigeminal ganglion (TG) cells from mixed nerves. Schwann cells (SCs) have emerged as valuable contributors in studies exploring the relationship between nerve cells and osteogenic differentiation. Serving as neurotrophic cells, SCs play a crucial role in providing nutritional support for axon regeneration and nerve cell growth. As glial cells in peripheral nerves, SCs are capable of secreting various neurotrophic factors, including nerve growth factor (NGF) and brain-derived neurotrophic factor (BDNF), and also release VEGF. These factors contribute to the intricate signaling network involved in bone physiology. Research involving SCs and their EVs has shown promising results. SCs and EVs have been found to induce enhanced ALP activity in osteoblasts, increase the number and size of calcified nodules, and result in deeper alizarin red staining. Furthermore, in mouse models, SCs and EVs have demonstrated a potential to assist in bone repair [173]. This suggests that the neurotrophic support provided by SCs, along with the release of bioactive factors, may have a positive impact on osteogenic differentiation and bone regeneration. The observed promoting effect may be attributed to the activation of specific signaling pathways, including TGF-β1 and Smad2 pathways [174]. The direct coculture of DRGs with MSCs has shown promising results in enhancing osteogenic differentiation. Zhang et al. [175] detected increased mRNA levels of ALP, OCN, OPN, and BMP-2 in the coculture group compared to MSCs alone [175]. However, a limitation was noted in that neural cells could only survive in osteogenic medium for about 5 days, preventing the tracking of the long-term effects of DRGs on various stages of MSCs osteogenic differentiation. To address the challenge of short-term survival, Silva et al. [176] designed a microfluidic device, enabling independent culture media for DRGs and MSCs while facilitating prolonged coculture. This extended coculture period allowed the observation of significantly upregulated early osteoblast-specific genes in the co-culture group. The study proposed that DRG neurons might promote the activation of the canonical Wnt signaling pathway in MSCs to enhance osteogenesis [176]. While the microfluidic device extends coculture duration, concerns are raised regarding its potential impact on the effectiveness of cell-secreted factors and EVs. This highlights the need for continued refinement and improvement in the coculture models of MSCs and DRGs to accurately capture the long-term effects and intricate signaling mechanisms involved in neuromusculoskeletal interactions. Future advancements in coculture techniques may provide more accurate insights into the sustained effects of DRGs on MSCs osteogenic differentiation.

### 3.5. Periodontal Cells

The unique anatomical structure of the maxillofacial region, particularly the jaw, poses distinct challenges and opportunities for reconstruction, given its frequent need for repair, especially in the alveolar bone. MSCs hold promise for transplantation around the alveolar bone to foster the regeneration and repair of periodontal tissue. Research has delved into the interactions involving oral bacteria, periodontal cells, and dental pulp cells in the context of jaw bone reconstruction. In the periodontal system, HGFs and periodontal ligament cells (HPLs) emerge as key players in periodontal tissue. Notably, in indirect coculture experiments with hMSCs, HPLs demonstrated an influence on the proliferation and osteogenic gene expression of MSCs. Specifically, HPLs increased the expression of leptin, although the concentration of leptin did not appear sufficient to impact osteogenic differentiation significantly. Additionally, HPLs were found to inhibit the mineralization induced by BMP-2 in hMSCs. These findings contribute valuable insights into the intricate interplay of various cell types within the periodontal system and their implications for bone regeneration in the maxillofacial region [177]. Among these cytokines, IL-1β, TNF-α, and periostin (POSTN) are implicated as potential contributors to this inhibitory influence [178]. In the context of indirect coculture experiments involving HGFs with hMSCs, there is observed inhibition in the expression of osteogenic genes in hMSCs, including OC mRNA and BMP-2 mRNA. Notably, IGF-1, secreted by HGFs, is suggested to play a role in this inhibitory process. However, the specific effects of other factors and the associated signaling pathways involved in this inhibition remain unclear. Unraveling the comprehensive network of factors and signaling cascades that modulate the crosstalk between HGFs and hMSCs in the context of osteogenic gene expression is crucial for a deeper understanding of the regulatory mechanisms at play in periodontal tissue. Further investigations are necessary to elucidate these additional factors and pathways, shedding light on the intricacies of the molecular interplay in the periodontal microenvironment during bone regeneration processes [179].

### 3.6. Multicell Mixed Culture

The aforementioned studies have predominantly concentrated on the impact of individual cell types on the osteogenic differentiation of MSCs. However, in natural biological processes, multiple cell types are intricately involved. Some researchers have initiated efforts to simulate the osteogenic differentiation of MSCs by coculturing various cell types in vitro. A relatively straightforward method involves directly extracting cells from the peripheral tissues of bones. In contrast to adipose-derived MSCs, which possess the capacity to differentiate into various cell types, the stromal vascular fraction (SVF) is a fraction formed by a combination of cells isolated from AT through enzymatic digestion and other methods. SVF comprises MSCs, EPCs, immune cells, smooth muscle cells, pericytes, and other stromal components such as platelet-rich plasma (PRP) [180, 181]. Choi et al. [182] conducted a study in which they cocultured SVF with MSCs in a Transwell penetrable cell culture chamber. Their findings revealed that the coculture induced the expression of osteogenesis-specific markers in the presence of BMP-2 and/or TGF-β1, within a short timeframe of 48 h. Notably, this induction was observed specifically for osteogenic markers and did not extend to markers associated with adipocytes and chondrocytes [182]. Totoliet al. [183] implemented a coculture system involving MC3T3-E1 cells with AT-MSCs and/or BM-MSCs using a similar coculture methodology. The outcomes of the study demonstrated that the ALP activity in the combined induction regimen, involving both MC3T3-E1 and MSCs, was slightly higher than that observed in the induction regimen with MC3T3-E1 cells alone [183]. Furthermore, they investigated the impact on bone repair by injecting AT-MSCs and/or BM-MSCs into rat skull defects. The study demonstrated that the combination of AT-MSCs and BM-MSCs exhibited an enhanced capacity for new bone formation. This observed synergistic effect could potentially be attributed to the divergent differentiation tendencies of MSCs derived from distinct sources. In another study by Jia et al. [184], a direct coculture approach was employed to coculture HSCs, MSCs, and EPC. The findings indicated that the coculture of MSCs + EPC + HSCs led to higher gene expression levels of COL1A, OPN, ALP, and OCN compared to both MSCs alone and MSCs + HSCs, suggesting a potential cooperative effect in promoting osteogenic markers [184]. Zhang et al. [173] conducted an experiment where they implanted a combination of SCs, intestinal epithelial cells (IECs), and IOBs into rat femoral defects using a β-tricalcium phosphate (β-TCP) scaffold. The results revealed robust fracture healing at 12 weeks, which was notably superior compared to groups involving IECs + IOBs and simple IOBs. Despite the positive outcomes, it is worth noting that the study did not delve into a detailed examination of osteoblast differentiation [173]. Zhang et al. [185] conducted a study where they indirectly cocultured DRG cells along with SCs derived from rat spinal cord, wrapping them around BM-MSCs. The results indicated an enhancement in the proliferation and multipotent differentiation ability of BM-MSCs in the coculture group. Notably, the study revealed that the maintenance of the stemness of BM-MSCs in this coculture setting was achieved through the upregulation of autophagy, specifically through the AMPK/mTOR pathway [185]. The outcomes of these experiments suggest the potential for simulating and enhancing the process of bone repair and possibly even bone formation in vivo through the coculture of multiple cells. However, it is essential to delve deeper into the specific factors and signaling pathways that promote osteogenesis in these coculture experiments. Additionally, the exploration of the types and proportions of various cells in coculture settings is necessary to gain a comprehensive understanding of the intricate mechanisms involved in bone regeneration. Further research and investigation are warranted to unravel the full complexities of these cellular interactions and optimize their application for therapeutic purposes.

### 3.7. Bacteria

In the body, particularly in infected wounds, MSCs come into contact with various bacteria, triggering a cascade of responses including inflammatory reactions, alterations in cell proliferation, apoptosis, and differentiation. Fiedler et al. [186] conducted a study comparing the impact of *Staphylococcus aureus*, *Streptococcus pyogenes*, and *Escherichia coli* on AT-derived MSCs (AT-MSCs) in the presence of their lipopolysaccharides (LPS) and/or teichoic acid (LTA). Notably, heat-inactivated Gram-negative *Escherichia coli* and LPS demonstrated the ability to induce both proliferation and osteogenic differentiation in MSCs. This effect may be attributed to the activation of the NF-κB pathway. Conversely, Gram-positive bacteria and LTA did not exert a significant influence on the osteogenic differentiation process [186]. *Salmonella typhi* (ST), a member of the Enterobacteriaceae family, is a gram-negative bacterium known as one of the pathogens causing human food poisoning. In a study by Mohamad-Fauzi et al. [187], ST was directly cocultured with AT-MSCs.

The findings revealed that AT-MSCs, when induced by ST treatment, exhibited heightened ALP expression, more pronounced alizarin red staining results, enhanced proliferation, and suppressed adipogenic differentiation and apoptosis [187]. *Porphyromonas gingivalis* (Pg), a pathogen associated with periodontitis, exerts varied effects on the biological activities of MSCs derived from different sources [188]. Pg-LPS has been observed to hinder the osteogenic differentiation of DPSCs by inducing inflammation. This is manifested through the upregulation of gene expression of inflammatory markers such as IL-1β, IL-6, and TNF-α, as demonstrated by Tang et al. [189]. Additionally, Tang et al. [189] investigated the impact of Pg-LPS at different concentrations on the osteogenic differentiation of BM-MSCs. Interestingly, at low concentrations, Pg-LPS was found to enhance both the proliferation and osteogenic differentiation of BM-MSCs. In contrast, at high concentrations, Pg-LPS induced apoptosis in these cells [189]. Regrettably, Pg exhibits poor adhesion and internalization to BMSCs, and there is limited research on the direct coculture of Pg and BMSCs in various proportions and its impact on osteogenic differentiation. Bacteria, including Pg, can also influence the immunomodulatory abilities of MSCs. It may be a promising approach to consider incorporating inactivated bacteria and their secretions into bone tissue engineering as a potential scheme for further exploration.

**Figure 3:**
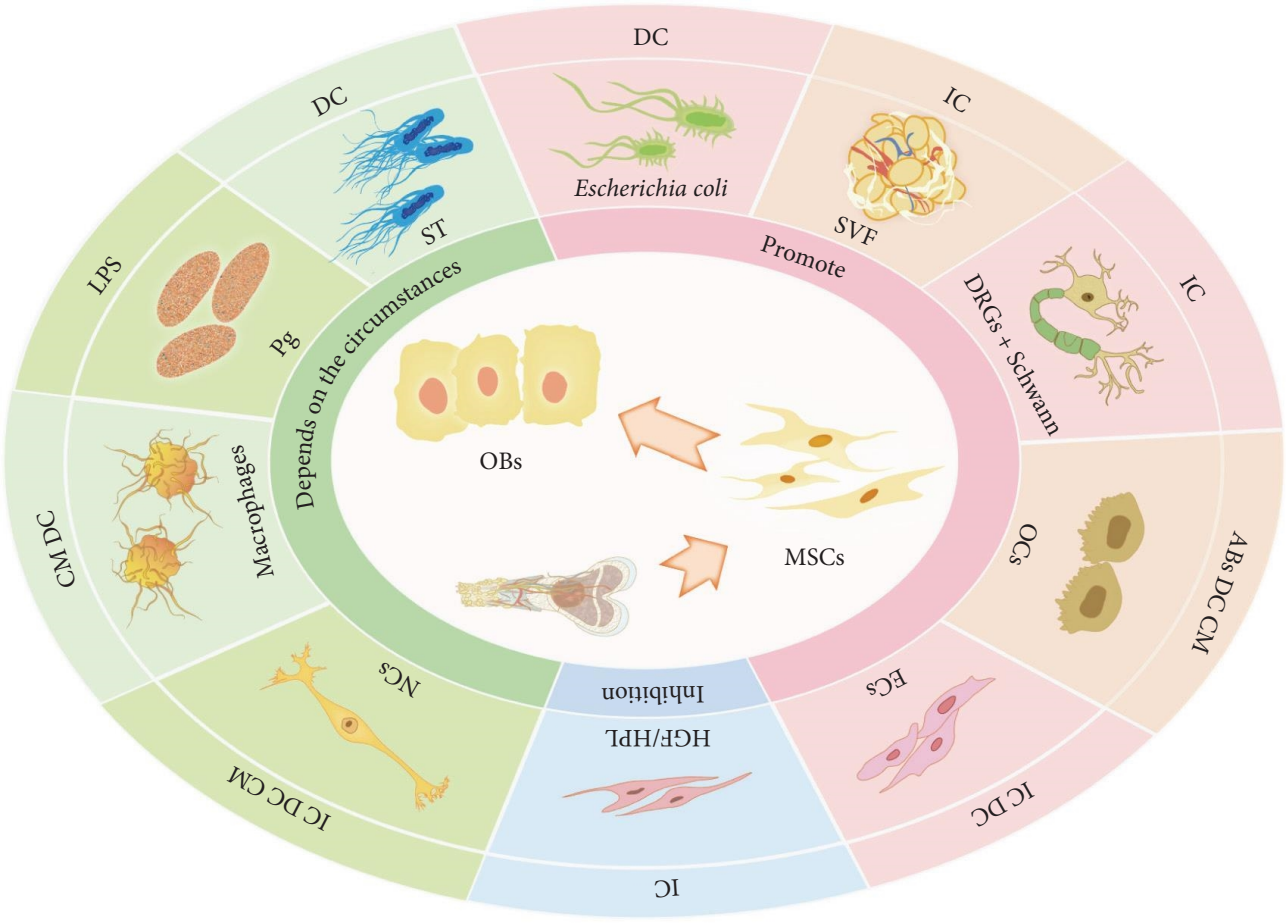
Effect of different cells on osteogenic differentiation of mesenchymal stem cells. ABs, apoptotic bodies; CM, conditioned medium; DC, direct coculture; DRGs, dorsal root ganglion neurons; ECs, endothelial cells; HGF, human gingival fibroblasts; HPL, human periodontal ligament cells; IC, indirect coculture; LPS, lipopolysaccharides; MSCs, mesenchymal stem cells; NCs, nerve cells; OBs, osteoblasts; OCs, osteoclasts; Pg, *Porphyromonas gingivalis*; Schwann, Schwann cells; ST, *Salmonella typhi*; SVF, stromal vascular fraction.

In addition to the abovementioned, various cells can affect the osteogenic differentiation of MSC (Table 1, Figure 3) drugs [190, 191], media [192], and transcription factors but also by various physicochemical properties including light [118], surface roughness [193], oxygen concentration, and tensile stress [158, 163, 164, 194].

## 4. Conclusion and Perspective

MSCs, recognized as one of the superior types of stem cells, hold immense potential in the field of bone tissue engineering and clinical applications. In the context of bone-related diseases, MSCs and their secretions have demonstrated significant efficacy, not only in experimental animal models but also in clinical settings. This includes successful outcomes in the treatment of conditions such as osteonecrosis [195, 196], cranial defects [197, 198], nonunion fractures [199, 200], and OA [201].

In recent years, researchers have employed various methods to induce the osteogenic differentiation of MSCs, aiming to explore more optimal induction protocols. During the development and differentiation of MSCs, a series of transcription factors and signaling pathways regulate this process. Among them, we consider Runx2 to be one of the most critical transcription factors, as it not only serves as a key marker in osteoblast differentiation but also acts as a pivotal hub influencing multiple signaling pathways. Any modulation or knockout of Runx2 could significantly impact osteogenesis. As research delves deeper, it has become apparent that individual signaling pathways or transcription factors do not simply exert a positive or negative influence on MSCs osteogenic differentiation. Even BMP-2, traditionally considered a positive osteogenic factor, can inhibit osteogenesis through the Notch pathway, while under certain conditions, the Notch pathway can promote osteogenesis. This leads us to believe that a precise positive/negative feedback mechanism exists within this complex regulatory network. Knocking down a single factor can disrupt normal cell differentiation, yet the overall regulatory balance may prevent significant phenotypic changes in bone formation. Moreover, this network is not limited to osteogenic lineage cells but is also linked to other organs, such as the gonads, highlighting the vast potential for further exploration in the regulatory factors influencing osteogenesis.

At the cellular level, the effects of single cells versus multiple cells on MSCs osteogenic differentiation differ considerably. These influences can be broadly categorized into two types: direct cell-to-cell contact, where binding of surface signaling molecules alters cellular behavior, including changes in secretion profiles and motility, and indirect cell interactions, primarily mediated by secretions. This can include the collection of conditioned medium, isolation of exosomes, induction of ABs, or the gathering of exotoxins to influence MSCs. The culturing methods for MSCs, whether 2D or 3D, also play a role. 3D cultures can be further divided into simple cell aggregates or scaffold-based complexes. These variable conditions complicate the understanding of how different cells affect osteogenic differentiation. Based on the information currently available, we believe that ECs, OBs, and SCs can positively influence osteogenic differentiation under these diverse conditions.

Despite extensive efforts by researchers to explore cocultivation of various cells with MSCs and delve into the associated mechanisms at the molecular level, there is still a substantial journey ahead. Firstly, current investigations into the influence of different cells primarily focus on the ultimate osteogenic outcomes, such as the quantity of calcium nodules and ALP staining at day 21 of induction differentiation. There is limited research exploring at which stage of osteogenic differentiation a specific cell exerts its influence. To address this gap, it may be possible to monitor different stages of MSCs osteogenic differentiation and replace cells within cocultivation chambers at various phases. Alternatively, the introduction of a self-apoptosis program through viral vectors within cells, triggered by specific cell factors produced during the MSCs osteogenic stage, could control the duration of cocultivation. Secondly, MSCs undergo the influence of multiple cells during the osteogenic differentiation process. While current studies often start with single-cell approaches, the interactions between different cell types should not be overlooked. Therefore, a future challenge lies in conducting precise multicell cocultures to better understand the intricate relationships between cells. Lastly, the control of cell ratios in multicell cocultures and the pathways through which cells influence each other are open questions. Future investigations could begin by varying cell proportions and analyzing osteogenic outcomes to further elucidate the mechanisms of influence. In essence, the field of MSC-based bone tissue engineering remains shrouded in uncertainty, with many aspects yet to be fully elucidated.

## Figures and Tables

**Table 1:** Effect of different cells on osteogenic differentiation of mesenchymal stem cells.

Cells		Types of MSCs			Way of influence	Result		References and remarks
Macrophage	M0, M1, M2	AT-MSCs			DC	+		[122]
						−		[136] (3D), [137] (3D)
		BM-MSCs			DC	+		[123] (3D), [124]*⁣*^*∗*^
					CM	−		[126]

Osteoclast	pOC, mOC	BM-MSCs			CM	+		[140, 141, 142]
		BM-MSCs			DC	+		[143] (3D), [144]*⁣*^*∗*^ (3D)
		BM-MSCs			ABs	+		[151]*⁣*^*∗*^, [153]*⁣*^*∗*^

Endothelial cells	EPCs	BM-MSCs			DC	+		[154]*⁣*^*∗*^, [156]*⁣*^*∗*^, [157]
					IC	+		[155]*⁣*^*∗*^, [157]
	HUVECs	BM-MSCs			DC	+		[158]
		UC-MSCs			IC	+		[159]
	HMECs	SHED			IC	+		[160]
		AT-MSCs			IC	+		[161]

Nerve cells	SCGs	BM-MSCs			IC	+		[171]*⁣*^*∗*^
	SH-SY5Y	BM-MSCs			CM	−		[172]
	SCs	BM-MSCs			DC	+		[173]*⁣*^*∗*^
		BM-MSCs			CM	+		[174]*⁣*^*∗*^
	DRGs	BM-MSCs			DC	+		[175]*⁣*^*∗*^, [176]*⁣*^*∗*^

Periodontal cells	HGF	BM-MSCs			IC	−		[179]
	HPL	BM-MSCs			IC	−		[177, 178]

Multiple cells	SVF	BM-MSCs			IC	+		[182]
	MC3T3-E1 + AT-MSCs	BM-MSCs			IC	+		[168]
	EPC + HSCs	BM-MSCs			DC	+		[184]
	DRGs + SCs	BM-MSCs			IC	+		[185]*⁣*^*∗*^
	SCs + IECs	BM-MSCs			DC	+		[173]

Bacteria	*Escherichia coli*	AT-MSCs			DC	+		[186]
	ST	AT-MSCs			DC	+		[187]
	Pg	DPSC			LPS	−		[188]
		BM-MSCs			LPS	±		[189]*⁣*^*∗*^

Abbreviations: 3D, 3D culture; ABs, apoptotic bodies; CM, conditioned medium; DC, direct coculture; DRGs, dorsal root ganglion neurons; EPCs, endothelial progenitor cells; HGFs, human gingival fibroblasts; HMECs, human microvascular endothelial cells; HPLs, human periodontal ligament cells; HUVECs, human umbilical vein endothelial cells; ID, indirect coculture; IECs, BM-MSC-derived induced endothelial cells; LPS, lipopolysaccharides; MC3T3-E1, mouse embryonic osteoblast; mOC, mature osteoclast; PC12, rat adrenal medullary pheochromocytoma; Pg, *Porphyromonas gingivalis*; pOC, preosteoclast; SCG, superior cervical ganglion; SCs, Schwann cells; SH-SY5Y, human neuroblastoma cells; ST, *Salmonella typhi*; SVF, stromal vascular fraction.

+, promote.

−, inhibition.

*⁣* ^*∗*^, Mouse bone marrow-derived mesenchymal stem cells.

## Data Availability

No new data were generated or used in this study.
